# Recalibration of perceived agency transfers across modalities

**DOI:** 10.1098/rsos.231962

**Published:** 2025-04-30

**Authors:** Belkis Ezgi Arikan, Kielan Yarrow, Katja Fiehler

**Affiliations:** ^1^ Experimental Psychology, Justus Liebig University Giessen, Giessen, Hessen, Germany; ^2^ Department of Psychology, City University of London, London, UK

**Keywords:** temporal recalibration, sensorimotor, agency, cross-modal

## Abstract

We experience our actions and their sensory consequences as synchronous despite small sensorimotor delays. This is attained by an adaptation process in which the sensorimotor system recalibrates temporal discrepancies between actions and their feedback, as long as causality is maintained (i.e. feedback follows action). Predictive motor mechanisms boost action–feedback binding, aiding in adaptation. Sensorimotor temporal recalibration is therefore closely linked with perceived control over the action and its sensory feedback (sense of agency, SoA). Interestingly, recalibration can also transfer to another sense, indicating a generalized mechanism that adjusts the timing of action–feedback events. It is unclear whether recalibration of perceived agency is driven by a similar mechanism. Here, we investigated cross-modal transfer of perceived agency and simultaneity in a sensorimotor recalibration task. In an adaptation phase, participants executed button presses leading to an immediate or lagged (150 ms) occurrence of a Gabor patch. Subsequently, they were asked to make simultaneity or agency judgements for action–feedback pairs (Gabor patch or tone) with variable response–stimulus asynchronies (RSAs). We found adaptation of synchrony and agency judgements with transfer of recalibration for agency judgements. Our findings suggest flexible recalibration of perceived agency, suggesting SoA is not inferred solely on a match with modality-specific motor predictions.

## 1. Introduction

Our actions and their sensory consequences are perceived to be temporally coherent, despite the existence of variable delays of neurophysiological and physical origin [1,2]. Although the auditory feedback when typing on the keyboard arrives some milliseconds later to the ears, the typing action and the associated auditory feedback is perceived as synchronous. Sensorimotor temporal recalibration describes the result of a compensatory process (i.e. a form of adaptation) in which actions and their sensory feedback become perceptually aligned in time when systematic temporal delays are present. Recalibrating the time of action–feedback events is crucial to maintain a coherent temporal perception of related events [3–6], as well as to feel in control of the sensory feedback generated by one’s own actions [7–9]. A multitude of studies suggest a related tendency to perceive one’s own actions and their sensory feedback closer in time than when similar sensory feedback is initiated externally [10–15]. It appears that voluntary actions may possess an advantage in structuring time (but see [16–18]).

Temporal recalibration is an established finding, but the fact that we can sometimes still experience asynchrony between our actions and their feedback, as in a badly synchronized video game, suggests that certain criteria need to be met to elicit recalibration. The presence and extent of sensorimotor temporal recalibration depends on causality; i.e. whether the two events are inferred to have a causal relation [19–21]. In the real world, effects follow their causes. Existing research indicates that the inferred causality between events influences how they are perceived in time, even when the physical temporal order is at odds with the inferred order [22,23]. In the case of voluntary actions, causality is established with greater ease: an action can only precede its effect [9,21]. Consequently, asynchronies between actions and accompanying sensations are more tolerated when the action precedes the sensory effect than when the action–effect order is reversed [20]. Once recalibration takes place, it is compelling, even leading to an illusory reversal of perceived action–feedback order, in which an immediate effect appears to precede its cause [6].

In addition to causality, existing work on sensorimotor temporal recalibration highlights the role of sense of agency (SoA), defined as the subjective experience of being in control of sensory events through one’s own action [9]. SoA involves both the attribution of authorship to a sensory event and the subjective experience of being in control of the sensory event [9,24]. It has been proposed that SoA operates at two distinct levels: a lower, pre-reflective level highlighted by an implicit sense of being in control (feeling of agency), and a higher, belief-like level underlined by an explicit sense of being in control (judgement of agency) [25]. The perception of synchrony between an action and a sensory event has been found to be stronger (i.e. to extend over a wider temporal window) when action–event order facilitates the SoA than when the sensory event precedes the action [21,26]. This highlights an elevated tendency to adapt perceived timing of actions to the subsequent sensory effects when one perceives authorship or control over the effect [27–31]. Relatedly, the perceived time of intentional actions and subsequent effects are attracted towards one another, known as intentional or temporal binding, which has sometimes been considered as an implicit measure of SoA [30], but see [16–18]. A comparator model has been proposed to account for SoA in which estimated sensory states associated with an action are compared with the actual sensory states resulting from the action. This is carried out by an internal forward model in which an efference copy of the motor command predicts the sensory states linked with the action [32]. In the case of a match, SoA over the sensory effect is inferred [33]. Multiple studies have provided evidence for the role of forward models in inferring SoA [6,10,14,30,34]. Recent work, however, challenges the role of the comparator model in inferring SoA and questions the assumption that temporal binding is an implicit measure of SoA. For example, audiovisual simultaneity around intentional actions is observed for passive movements of the limb [35] or even in the absence of an action (passive viewing) [36]. These results are rather in line with the notion that SoA results from multisensory cue integration in which actions and accompanying sensations are integrated into a single percept whenever a common cause can be established [18,37]. Additionally, some studies have demonstrated that implicit and explicit measures of SoA, such as temporal binding and agency judgements, are not correlated [38,39], but see [40]. Yet other studies point to potential confounds in experimental conditions used to assess SoA in conventional paradigms [16,17]; for example, differences in attentional load between experimental conditions involving actions and sensory stimuli, and baseline conditions involving only one of these events [17,41].

Despite the controversy regarding temporal binding and SoA, timing between actions and sensory events provides a useful cue to perceived agency [19,21,26,42]. In the case of sensorimotor recalibration, there is an obvious link between time perception and SoA when dealing with temporal discrepancies. Nevertheless, existing work also points to a dissociation between the two [20,43]. Rohde *et al*. [21] investigated the relationship between time and perceived agency for button presses causing visual flashes in which they asked participants to judge either the simultaneity between the two events or their feeling of control over the flash via the button press. They found that the time window in which the two events are judged as simultaneous is narrower compared with when participants judged control over the flash through their button press. A similar result was observed by Bonnet *et al*. [44], who found this difference to be enhanced when the sensory event (coherent motion onset) was left/right congruent with the hand used for the action. In another study, Sugano [43] found differences in the recalibration of timing and perceived agency for button press–tone events. More specifically, systematic temporal delays between button presses and subsequent tones led to both a shift in the point of subjective simultaneity and a widening of the window of perceived simultaneity for the events. Recalibration of agency judgements was weak and associated with a decreased tendency to respond as not being in control of the feedback rather than a decreased sensitivity to delays when judging agency. These results suggest a potential dissociation between time perception and SoA arising from adaptation. More specifically, recalibration of perceived agency seems to be less sensitive to systematic action–feedback delays compared with the recalibration of synchrony.

The differences that emerge when recalibration is measured via judgements about time versus SoA can be scrutinized by examining their proposed underlying mechanisms. Classically, sensorimotor temporal recalibration is measured via timing judgements, and observed following lag adaptation (i.e. use of delayed feedback). Because this recalibration can transfer across sensory modalities ([4,26,27]; but see [28,29] and different limbs (hand and foot tapping) [45]), its origin may be a supramodal time-perception module (i.e. supramodal clock) for actions and their sensory feedback. Such a supramodal recalibration might be beneficial in compensating for delays due to neural transmission or processing demands the nervous system is faced with [1,2,46].

It is possible that, like perceived timing, the SoA also depends on an assessment about timing derived from the same supramodal clock, and would thus show similar recalibration following adaptation. However, we have already noted that SoA has been hypothesized to reflect the outcome of a comparator process when an internal forward model is used to compare predicted and actual sensory feedback associated with the movement. Forward models are highly specific; they make specific predictions with regard to the temporal and spatial characteristics of the action–feedback event and about the identity of the action’s feedback (e.g. a visual effect of certain intensity and duration) [27,47]. This might thus imply no cross-modal transfer for the recalibration of perceived agency. However, there is also evidence that the SoA is more flexible to mismatches between the predicted and actual feedback [42,48]. For example, Desantis *et al*. [42] demonstrated that sensory feedback identity does not seem to drive temporal binding of action–feedback events [49]. This finding undermines the importance attributed to the comparator process in establishing SoA and highlights a more flexible mechanism. Similarly, Moore & Haggard [48] suggest that judgements of perceived agency result not only from motor prediction, but also from an inferential mechanism, i.e. when reliability of predictions is low, SoA can be inferred postdictively as long as an action–feedback association can be made. The latter proposal is also in line with the notion that recalibration reflects multisensory integration of cues arising from a common origin [18,19]. Together, these findings indicate both similarities and dissociations between timing and SoA and, in particular, for the stimulus-specificity of their comparative temporal recalibrations.

In this study, we aimed to investigate the relationship between temporal recalibration and perceived agency, specifically perceived authorship, for action–feedback events measured by judgements of synchrony or perceived agency. Assuming that the action–feedback identity may be the dissociation point for the temporal recalibration of timing versus agency judgements, we asked, to our knowledge for the first time, whether recalibration of perceived agency could generalize to another feedback modality. If the perceived action–outcome association is mainly modulated by their temporal order, due to a common cause assumption [19], we would expect recalibration of agency judgements to transfer to another modality. Alternatively, if a comparison with the specific identity of the sensory feedback predicted by a recalibrated forward model mainly drives agency judgements, a change of modality would render the recalibrated forward model invalid. The brain might then revert to a different (unadapted) model better aligned to the identity of the feedback, and there should be no transfer. The latter prediction would indicate a dissociation between perceived timing and agency judgements in terms of the mechanisms involved. A second objective was to investigate cross-modal recalibration using a much larger sample size than previous studies had used (*ca n* = 10) to obtain more accurate estimates, since cross-modal transfer is weaker than within-modality recalibration [6,50–53].

To this end, we ran an experiment in which the participants performed button press actions that resulted in visual feedback (a Gabor patch). We investigated cross-modal transfer from vision to audition, since it shows stronger transfer effect than transfer from audition to vision [51]. In order to induce temporal recalibration, we inserted systematic delays (lags) of either 0 or 150 ms between button press–visual events (adaptation phase). We then tested for recalibration by presenting button press–visual events with variable response–stimulus asynchronies (RSAs) and asking the participants to judge simultaneity between them (test phase). To examine the transfer effect, we presented button press–auditory events with variable RSAs after an adaptation phase of button press–visual pairs, in a separate block. Regarding recalibration of agency judgements, we repeated both of these blocks in a similar fashion, this time asking whether the participants felt in control over the Gabor patch (or the tone in the transfer block) through their button press. Here, our focus was on the perceived authorship attribution of SoA.

## 2. Material and methods

### 2.1. Participants

The experiment was approved by the local ethics committee (Lokale Ethik-Kommission des Fachbereichs 06; LEK-FB06) and was performed in accordance with the Declaration of Helsinki except for preregistration [54]. Our sample included 52 university students (37 females, 24 ± 3 years). They provided written informed consent prior to participation. Data from two participants were excluded due to technical failure. An additional two participants dropped out of the study without completing either of the two tasks (agency or simultaneity), resulting in a final sample of 48 participants (36 females, 24 ± 3 years), 46 of whom completed all eight blocks (one completed six blocks, one four blocks). Data of four from eight blocks were excluded from one further participant (see §2.6). All participants were right-handed as confirmed by the Edinburgh handedness inventory (EHI score 88.08 ± 21.82) [55]. They reported normal or corrected-to-normal vision, and normal hearing. In addition, none reported having current psychiatric or neurological conditions or taking related medication. Participants received monetary compensation for their participation.

### 2.2. Sample sizes

Sample size was determined based on our previous study in which we investigated within (*t*‐test, Cohen’s *d* = 0.83) and cross-modal (Cohen’s *d* = 0.57) adaptation effects [51] for motor–visual events. Considering the possibility of higher variability for agency judgements [21], we assumed a minimum effect size of interest of Cohen’s *d* = 0.5. In order to detect a Cohen’s *d* of 0.5 (with alpha = 0.05 and power = 0.90, two-tailed *t*‐test), a minimum sample size of 44 is necessary. We recruited 52 participants in total. Considering the excluded participants and the participants that partially completed the blocks, a final sample size of between 45 and 48 (depending on the exact contrast, as not all participants completed all conditions) was in all cases appropriate to provide greater than 90% power.

### 2.3. Stimuli and apparatus

Visual stimuli consisted of Gabor patches (1.49°, spatial frequency = 5 cycles/degree, duration = approx. 33.4 ms), and were presented on a 24″ computer monitor (Viewpixx 3D, 1920 × 1080 pixels resolution, 120 Hz frame refresh rate). Auditory stimuli were brief sine-wave tones (frequency = 2000 Hz, duration = approx. 33.4 ms with 2 ms rise/fall slopes), and were presented via headphones. Stimulus presentation and response recording were controlled by Matlab 2019a (The MathWorks Inc.) and Psychtoolbox−3 [56,57]. Participants’ yes/no responses were recorded via a keyboard (‘V’ and ‘N’ buttons on the keyboard).

Prior to the experiment, we measured the internal delay between the keypresses and the auditory/visual stimuli. The internal delay between a keypress and a visual stimulus was 30.28 ± 4.39 ms, and a keypress and tone was 41.79 ± 4.14 ms.

The experiment was conducted in a dimly lit room. Participants sat at a desk in front of a monitor with a viewing distance of approximately 55 cm. Their right index finger was placed on a left key of a mouse pad (Perixx Peripad-504) that was used to trigger visual and auditory stimuli. The stimuli were presented only when the left key on the mouse pad was pressed down completely. The mouse pad was placed in a custom-made box to prevent the participant from using visual cues from their hand to perform the task. The bottom of the box was covered with a cushion to ensure a comfortable hand/forearm positioning. White noise was presented throughout the experiment to mask any auditory cues. In addition, we used sound attenuating headphones in order to prevent any possible sounds from the outside. Prior to the experimental blocks, we made sure that the participants could hear the tones clearly.

### 2.4. Experimental design

The experimental design consisted of three within-subject factors; namely, task, sensory modality and adaptation delay. The first factor *task* corresponded to the type of judgement the participants had to make following adaptation, and consisted of simultaneity and agency judgements. The second factor *sensory modality* comprised the modality of the sensory feedback in the test phase, and could be either visual or auditory. The third factor, *adaptation delay*, described the lag between the action and the sensory feedback at adaptation, and could either be 150 or 0 ms (not considering the internal delay of the system).

The dependent variables were simultaneity and agency judgements for keypress–stimulus pairs (Gabor or tone) with 15 RSAs (−333, −250, −133, −100, −66, −33, 0, 33, 66, 100, 133, 250, 333, 417, 500 ms) inserted between the keypress and the stimulus presented in the test phase. Negative values correspond to trials in which the sensory stimulus was presented before the keypress. The RSA range and step sizes were based on a pilot study in which we aimed to optimize the experimental design (e.g. number of adaptation and test trials, range of RSAs, number of repetitions per RSA) for assessing agency and simultaneity judgements. For trials in which the stimulus preceded the keypress, we presented the stimulus based on an estimation of when the participant would press the key. More specifically, we estimated the keypress time of the participant on a trial-by-trial basis by calculating the median keypress time of the previous five keypresses. Nevertheless, as early sensory stimuli can trigger keypresses [9], we calculated and used the actual RSAs for trials in which the stimulus was presented before the keypress (see §2.6).

Each participant attended four experimental sessions, completed over four days. In each session, they completed two blocks of different experimental conditions, varying the sensory modality at test. Levels of the factor adaptation delay were presented on separate days in order to prevent possible carryover effects between adaptation delays [49,50]. Moreover, the two judgement tasks were introduced on separate days, namely on the first and on the third day, after the completion of the initially assigned judgement task. We decided to introduce these tasks one after the other, because in the pilot experiment participants had difficulties in distinguishing between these judgements, especially if both tasks were presented on the same day. Levels of the factor sensory modality were presented in separate blocks, so were fully predictable. To minimize the potential influence of temporal event order on agency judgements in the stimulus–action trials [9], we told participants that either the computer or their button press could initiate visual or auditory feedback in the SoA condition. The order of conditions was pseudorandomized across participants.

### 2.5. Procedure

**Figure 1.**
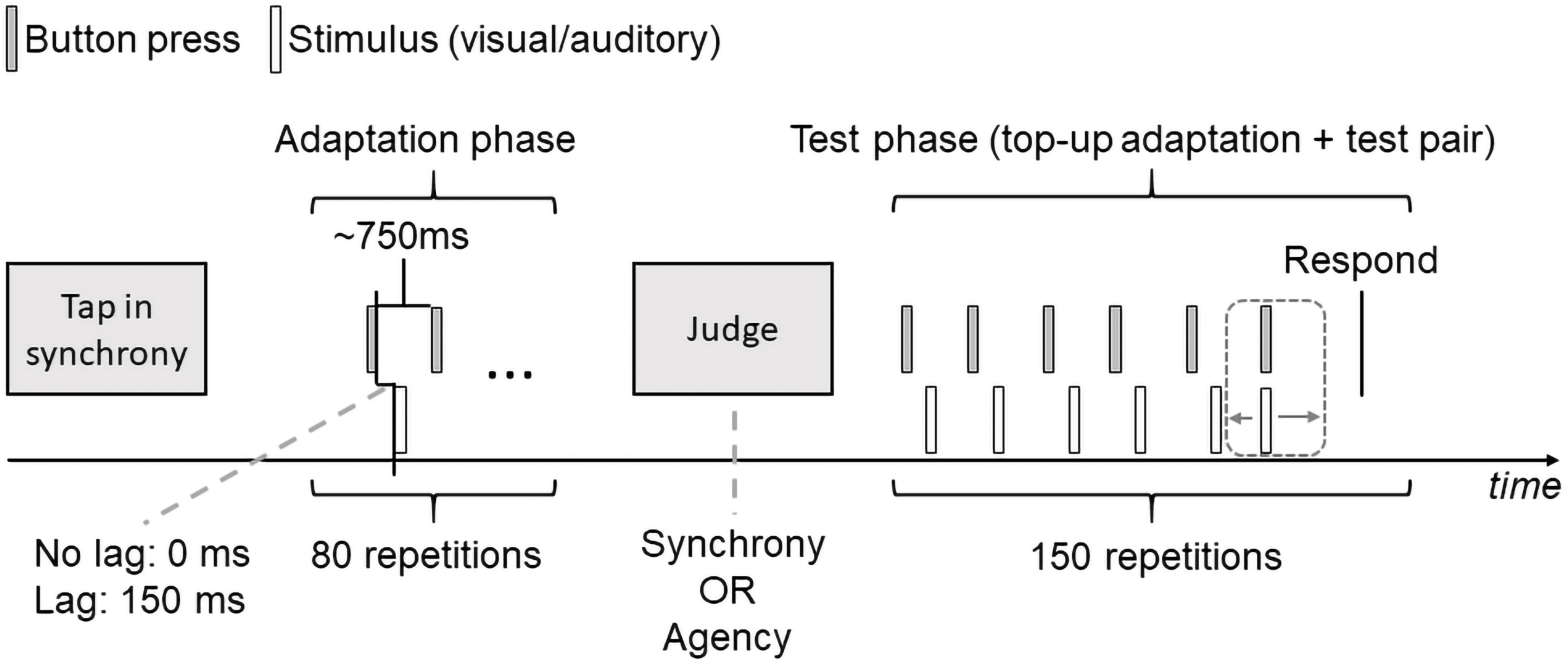
Schematic of an experimental block. Each block consisted of 80 adaptation pairs followed by 150 top-up adaptation + test pairs. Participants were instructed to perform button presses each of which were followed by a Gabor patch with (150 ms) or without (0 ms) a systematic lag between the two. The test phase consisted of five top-up adaptation pairs that involved button press–Gabor pairs plus a test pair. Stimulus modality in the test pair could be visual (Gabor) or auditory (tone), and involved variable RSAs between the button press and the stimulus pair. Immediately after the test pair, participants were asked to judge whether the test pair were synchronous or whether they thought they caused the stimulus through their button press (Respond).

A schematic of an experimental block is shown in figure 1. Each experimental block consisted of an adaptation phase and a test phase. The adaptation phase consisted of 80 trials of keypress–Gabor patch pairs whereas the test phase consisted of 150 trials, each of which involved five top-up adaptation pairs consisting of keypress–Gabor pairs as in the adaptation phase and one test pair. Each block began with the adaptation phase. Participants were instructed to perform keypresses at a constant pace, targeting an inter-tap interval of 750 ms. Each keypress led to the occurrence of a Gabor patch in the middle of the screen, either immediately (0 ms lag) or after a 150 ms lag. We informed the participants about their keypress pace after every five keypress–Gabor pairs: if the median interval between keypresses was below 700 ms or above 800 ms, they received ‘Keypress: slower’ and ‘Keypress: faster’ instruction presented for 750 ms on the computer screen, respectively.

After the adaptation phase, the test phase began. An instruction prompted the participants to perform keypresses, for six times on each trial, still keeping the instructed pace. The first five events consisted of keypress–Gabor pairs as in the adaptation phase (top-up adaptation). In separate blocks, the sixth keypress was accompanied by either a Gabor patch or a beep, with variable RSAs between the keypress and the stimulus (selected at random from the set of 15 possible RSAs, with each RSA repeated 10 times). After a 500 ms interval, the question ‘Synchronous?’ or ‘Self-initiated?’ (i.e. did you initiate the stimulus via your button press?) appeared on the screen depending on the experimental block. Participants used the keys ‘V’ and ‘N’ for ‘Yes’ and ‘No’ to provide their responses within a response period of 2000 ms. Immediately after the response period, an inter-trial interval ranging from 500 to 1500 ms followed. At the end of the trial, the participants received feedback about their keypress pace on that trial. If the median keypress interval was below 650 or above 850 ms, participants received ‘Slower’ and ‘Faster’ instructions, respectively. These slow and fast trials were rejected and repeated in a subsequent trial, until it fell within the expected keypress pace. Moreover, for all trials, we allowed a maximum button press interval of 850 ms between the fifth and the sixth button presses. We rejected and repeated those trials in which these criteria were not met.

Prior to the experimental blocks, participants practised the keypress pace, by tapping in synchrony with a metronome for 3 min. They were given feedback on their performance, and offered additional training if they felt they needed more practice. All participants were able to tap in accordance with the auditory signal in the initial training. After the tapping practice, the participants received a short training block for the upcoming experimental block. We encouraged participants to take breaks between the blocks, but did not force a fixed break. Each experimental block lasted for approximately 25 min. If the total duration of a given block exceeded 35 min due to repetitions, we concluded the block, as this would result in deterioration of time perception [58]. The total duration of the experiment over the four sessions was approximately 4.5 h.

### 2.6. Data analysis

At zero and positive RSAs, the stimulus was triggered from the response and therefore matched the intended timing (aside from software delays). Typically, 90 such trials per block were entered into the analysis (across the nine positive RSAs) with a small number of truncated blocks containing substantially fewer trials (5% showing greater than 20% reduction, minimum of 19 trials). For the 60 remaining trials per block, with RSAs intended to be negative (based on the expected time or response), we attempted to determine the true RSA (binned to the nearest screen refresh) for use in the analysis. Due to a coding error only a subset of these estimates (median 27%) could be entered into the analysis. We calculated the proportion of ‘yes’ responses at each RSA and in each condition. We removed the agency judgement data from one participant because of too few yes responses (less than 2% of trials).

Traditionally, data from this kind of experiment are fit separately for each condition and each observer (resulting in our case in 2 × 2 × 2 × 48 = 384 separate psychometric function fits). Then, one or more of the derived model parameters from each fit are compared across conditions using a subsequent inferential test such as ANOVA. However, that approach fails to capitalize on all the information in the combined dataset. Here, we fit a single Bayesian multi-level model to all participants and conditions at once using the Stan programming language interfaced from R [59] via the RStan package [60]. This multi-level approach is (in principle) an all-in-one implementation of the traditional analysis pipeline, using a similar number of parameters to characterize the data and draw inferences. Among other benefits, it allows to incorporate data from the three participants with missing conditions, and to appropriately weight cases with reduced numbers of trials.

We first attempted to fit the model using four chains (i.e. four independent parallel samplers evaluating different parameter values) each exploring the likelihood surface via the default Hamiltonian Monte Carlo no-U-turn sampling (HMC NUTS) algorithm. This algorithm is intended to retain samples in proportion to the height of the posterior distribution, and thus estimate it. Regrettably, a complex and presumably multi-modal posterior meant that we could not achieve good mixing between chains, precluding the derivation of valid Bayesian credible intervals. We therefore opted to instead obtain a maximum-likelihood fit, searching the parameter space from multiple starting points via Stan’s L-BFGS quasi-Newton algorithm. For (non-parametric) statistical inference and estimation of standard errors (used to calculate confidence intervals and effect sizes) we programmed permutation tests and bootstrap procedures, re-fitting the model from each of 999 random permutations (across all cells of the design within each participant) or re-samples (re-sampling complete participants with replacement), respectively. With 999 permutations, the 95% confidence interval around *p* = 0.05 is 0.014, so we report *p*-values in the range 0.036−0.064 as having ‘marginal’ significance.

We fit an adapted version of the multi-level At-A-GLANCE model [61]. This model posits signals of two events (such as an action and its consequence) propagating towards a decision hub, each having Gaussian latency noise. Their subjective difference in arrival times is then categorized using a pair of decision criteria that vary randomly from trial to trial. Multi-level models add a set of group-level parameters to a heterogeneous foundation (essentially, a single-level model fitted to each participant). In this case, the heterogeneous foundation specifies a binomial distribution for the number of yes responses ($Y_{∆tijk}$) from each participant in each cell of the design (i.e. at the *i*th level of task, *j*th level of sensory modality and *k*th level of adaptation) and each response–stimulus asynchrony (∆t),


(2.1) $$Y_{∆tijk}\sim B\left(N_{∆t},l+p_{∆tijk}-2lp_{∆tijk}\right),$$

where there are $N_{∆t}$ trials at each RSA (per condition), l is a free parameter representing (half) the lapse rate with which a participant is distracted and therefore guesses a response (assumed identical across all conditions) and


(2.2) $$p_{∆tijk}=\Phi \left[\frac{∆t-\tau_{ijk}-{∆\delta }_{ijk}/2}{\sigma_{Lijk}}\right]-\Phi \left[\frac{∆t-\tau_{ijk}+{∆\delta }_{ijk}/2}{\exp\left(m_{ijk}\right)\sigma_{Lijk}}\right].$$

In equation (2.2), exp is the exponential function and Φ is the standard normal cumulative distribution function. The remaining four symbols ($\tau_{ijk}$, ${∆\delta }_{ijk}$, $\sigma_{Lijk}$ and $m_{ijk}$) are terms constructed from a set of free parameters as described next,


(2.3) $$\tau_{ijk}=\bar{\tau }+\;t_{i}\beta_{\tau t}+s_{j}\beta_{\tau s}+a_{k}\beta_{\tau a}+t_{i}s_{j}\beta_{\tau ts}+t_{i}a_{k}\beta_{\tau ta}+s_{j}a_{k}\beta_{\tau sa}+t_{i}s_{j}a_{k}\beta_{\tau tsa},$$

(2.4) $$\Delta \delta_{ijk}=\bar{\Delta \delta }\exp\left(t_{i}\beta_{\delta t}+s_{j}\beta_{\delta s}+a_{k}\beta_{\delta a}+t_{i}s_{j}\beta_{\delta ts}+t_{i}a_{k}\beta_{\delta ta}+s_{j}a_{k}\beta_{\delta sa}+t_{i}s_{j}a_{k}\beta_{\delta tsa}\right),$$

(2.5) $$\sigma_{Lijk}=\bar{\sigma_{L}}\exp\left(t_{i}\beta_{\sigma t}+s_{j}\beta_{\sigma s}+a_{k}\beta_{\sigma a}+t_{i}s_{j}\beta_{\sigma ts}+t_{i}a_{k}\beta_{\sigma ta}+s_{j}a_{k}\beta_{\sigma sa}+t_{i}s_{j}a_{k}\beta_{\sigma tsa}\right),$$

(2.6) $$m_{ijk}=\bar{m}+\;t_{i}\beta_{mt}+s_{j}\beta_{ms}+a_{k}\beta_{ma}+t_{i}s_{j}\beta_{mts}+t_{i}a_{k}\beta_{mta}+s_{j}a_{k}\beta_{msa}+t_{i}s_{j}a_{k}\beta_{mtsa}.$$

In equations (2.3)–(2.6), bar notation (e.g. $\bar{\tau }$) is used to describe grand mean parameters (across conditions), $t_{i}$ is the effects-coded value for the ith level of task (and is thus substituted with ±0.5 depending on the task), $s_{j}$ is the effects-coded value for the jth level of sensory modality, $a_{k}$ is the effects-coded value for the kth level of adaptation, and the seven β coefficients describe, depending on their subscripts, main effects for *t*ask, *s*ensory modality, *a*daptation and their two-way (ts, ta, sa) and three-way (tsa) interactions. That makes 33 free parameters for each of 48 participants; a total of 1584 for the group as a whole.

In this model, the individual τ and ∆δ parameters capture the midpoint and width (respectively) of each participant’s psychometric function. They provide an alternative (and mathematically equivalent) way of describing the positions of two decision criteria (because ∆δ is the distance between these criteria, which are centred on τ). The $\sigma_{L}$ and m parameters describe noise affecting the left flank of the psychometric function, and the noisiness of the right flank relative to the left flank (m of 0 indicating an identical magnitude of noise), respectively. β coefficients describe changes in these parameters across conditions. The multi-level model additionally estimated random variation across the group via group-level distributions from which the individual-level parameters were drawn. This required a further 65 parameters. For example, we estimated, for the Gaussian group-level distribution of individual $\overset{-}{\tau }$ parameters, a group mean ($\mu_{\tau }$) and standard deviation ($\sigma_{\tau }$). Similarly, for the group-level distribution of the main effect of task upon τ ($\beta_{\tau t}$), we estimated a further group mean ($\mu_{\tau t}$) and standard deviation ($\sigma_{\tau t}$). In all, there were 1649 parameters, fitted using 6617 binomial data points.

We evaluated the fit of individual participants in a manner akin to calculating a Bayesian *p*-value [62] representing the proportion of posterior samples for which the likelihood of each participant’s actual data was lower than that for a random binomial draw conditioned on model parameters. However, we used the maximum-likelihood estimation (MLE) fit in place of posterior samples, forming a null distribution via 1000 random draws. If the model is correct for an individual, the resulting overdispersion value should be around 0.5, with higher values indicating overdispersion and therefore a potentially incomplete or erroneous model. The data and analysis scripts are available at https://osf.io/hy7k8/.

## 3. Results

### 3.1. A simple decision process could be used to classify response–stimulus asynchronies

**Figure 2.**
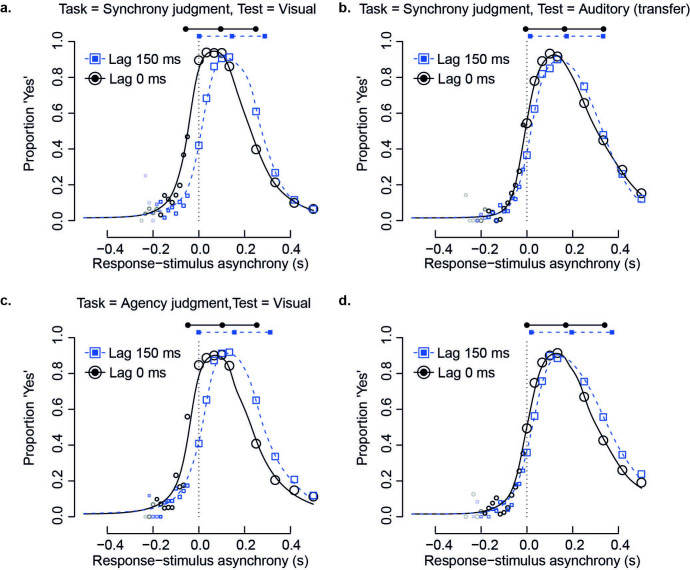
Group-average raw data and model fit. Size of open symbols indicates the average number of trials included in the analysis per participant. Opacity of open symbols indicates the number of participants contributing to a data point. Each plot contrasts baseline (black circles) with lag-adapted conditions (blue squares). Filled symbols above plots illustrate how model parameters describe the midpoint and extent of the psychometric function. (a) Synchrony judgements with visual test stimuli. (b) Synchrony judgements with auditory test stimuli, testing transfer of timing recalibration. (c) Agency judgements with visual test stimuli. (d) Agency judgements with auditory test stimuli, testing transfer of perceived agency recalibration.

We derived summary measures of behaviour for both simultaneity and agency judgements by fitting a Bayesian multi-level implementation of an established observer model of the simultaneity–judgement task [61,63]. The model assumes that observers have access to a noisy impression of the asynchrony on each trial (the subjective asynchrony) which they classify using decision criteria (which are also noisy) to form a binary response. Figure 2 shows the mean raw data from the entire group in all eight conditions, along with the (equivalently averaged) psychometric function predicted by the model (separate individual data and predictions for each participant can be found in electronic supplementary material, figure S1). Data points lie close to model predictions, indicating a good fit. This observation is supported by considering the magnitude of residual errors relative to what should be expected if data are binomially distributed around model predictions. We quantified this by comparison with the simulated null distribution, with values exceeding 0.95 (indicating significant overdispersion) observed for only 1/48 participants. Given that models are always approximations of reality, this is a fairly stringent test for the adequacy of model fit. The distribution of overdispersion values (mean 0.24, s.d. 0.27) is shown in electronic supplementary material, figure S2.

### 3.2. Recalibration and transfer of recalibration occur, but to different extents

It is common to report points of subjective simultaneity (or perceived agency) derived from the central tendency of the relevant psychometric function as the primary measure of temporal recalibration. Our multi-level model captured psychometric central tendency for each individual via a parameter termed τ. Formally, this indicates the midpoint between two decision criteria, which together define the region of subjectively perceived asynchrony that gets translated into the categorical response ‘yes’. To model changes across conditions, τ was combined with a set of modifying parameters ($\beta_{\tau }$ coefficients) to generate a separate estimate in each cell of the design. Variation across the group was also modelled, i.e. the model is similar to a generalized linear mixed model, with both fixed and random effects and the use of effects coding for all factors, but predicts a bespoke psychometric function appropriate for the current tasks. The resulting estimates for group-mean central tendency in different conditions are shown above each panel from figure 2 as the middle one out of three filled symbols. A further model parameter (∆δ) describes the width of the psychometric function (formally, the distance between the aforementioned decision criteria). It is represented in figure 2 by the length of the line connecting the outer two filled symbols above each plot.

**Figure 3.**
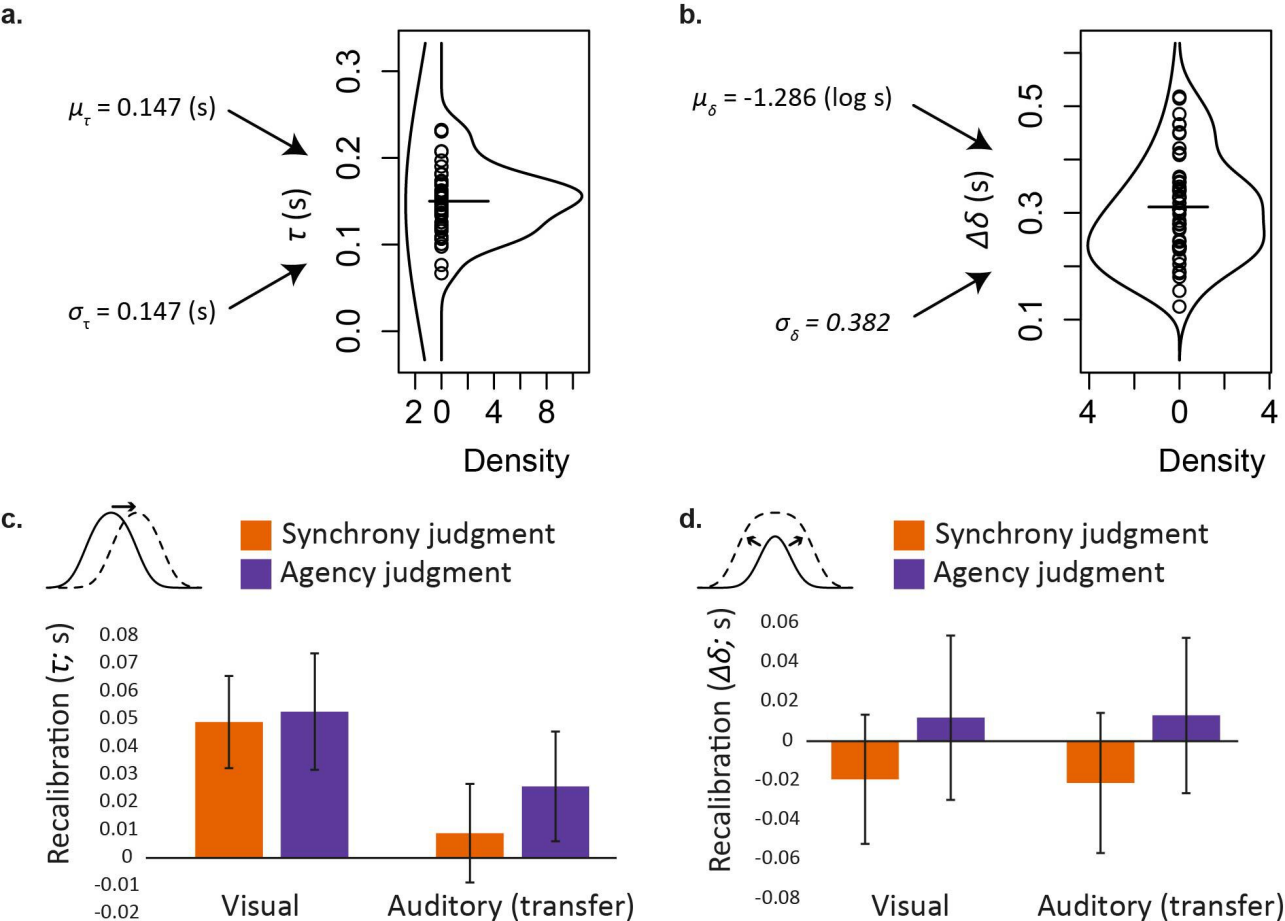
Effects of lag adaptation on summary measures for psychometric-function central tendency and width. (a) Parameter $\overset{-}{\tau }$, which describes the average central tendency of the psychometric function across all experimental conditions. Values of $\mu_{\tau }$ and $\sigma_{\tau }$ predict variation in τ across the population (hourglass plot, left lobe). Within the hourglass plot, individual estimates of $\overset{-}{\tau }$ are shown as black circles. Their mean appears as a solid horizontal line, and a kernel-density estimate of their distribution completes the hourglass plot as the right-hand lobe. (b) Group-level parameters $\mu_{\delta }$ and $\sigma_{\delta }$, which describe the (lognormal) distribution of the participant-level parameter $\overset{-}{\Delta \delta }$. This in turn describes the average width of the psychometric function across all experimental conditions. Format otherwise as per part a. (c) Differences in psychometric function central tendency (τ) between lag 150 and lag 0 conditions (i.e. recalibration effects) plotted separately for each combination of task with each test-stimulus modality. Error bars show 95% confidence intervals on these differences. (d) Adaptation-induced differences in psychometric function width (Δδ). Format otherwise as per part c.

Estimates of τ for each cell of the design were assessed via a permutation approach, conceptually similar to a 2 x 2 x 2 repeated-measures ANOVA. On average (across the two different tasks and two different modalities of test stimuli) the psychometric function shifted rightwards in lag 150 conditions relative to lag 0 conditions (main effect of adaptation of 34 ms, Cohen’s *d* = 1.07, *p* ≤ 0.001). This indicates that our participants experienced temporal recalibration. Furthermore, on average (across the two different tasks and two different lags) the psychometric function was centred at more positive values when the test stimulus was auditory rather than visual (main effect of test 51 ms, *d* = 1.04, *p* ≤ 0.001). This indicates that sounds had to lag actions more than lights in order to be perceived as synchronous with, or caused by, those actions, perhaps because propagation latencies are longer for visual compared with auditory stimuli. An average difference was also marginally evident between simultaneity and agency judgements, with more positive (i.e. test-lagging) estimates of central tendency for agency judgements (main effect of task 11 ms, *d* = 0.30, *p* = 0.061). This suggests that a slight addition to the large delay between action and outcome that maximized ‘yes’ responses (see next paragraph) was preferred to infer SoA, relative to judgements about simultaneity. However, these average differences were tempered by statistically significant interactions between experimental factors, which are illustrated in figure 3.

The upper panels of figure 3 provide a sense of individual variation in the central tendency (τ) and width (∆δ) of the psychometric function. They also illustrate how this variation was modelled by estimating the parameters of appropriate group-level distributions (e.g. $\mu_{\tau }$ and $\sigma_{\tau }$ for the distribution of $\overset{-}{\tau }$). Panel a illustrates that psychometric-function central tendency averaged across all conditions ($\overset{-}{\tau }$) had a mean of 150 ms. This implies that, in general, test stimuli that followed the button press were most likely to be judged simultaneous with, or caused by, the action. Delayed awareness for actions is not uncommon when using category matching tasks with yes/no response options, such as the simultaneity judgement task [44,45] and is also observed in synchronization tapping tasks [49,64].

More importantly for current purposes, the lower panels of figure 3 show the effects of lag adaptation in different conditions, again focusing on the central tendency (τ, panel c) and width (∆δ, panel d) of the psychometric function. For central tendency (figure 3c), recalibration was significant for most combinations of task and test stimulus (pairwise contrasts: visual synchrony, *d* = 0.86, *p* ≤ 0.001; auditory synchrony, *d* = 0.15, *p* = 0.391; visual agency, *d* = 0.73, *p* ≤ 0.001; auditory agency, *d* = 0.38, *p* = 0.015). This result addresses one of our core aims, which was to determine for the first time whether recalibration would show any cross-modal transfer when measured using agency judgements—it does. However, recalibration was greater (on average) with visual test stimuli (left-hand bars) than for auditory transfer stimuli (right-hand bars; two-way interaction, *d* = 0.52, *p* = 0.002), so transfer was incomplete. Hence, overall, changes in the central tendency of the psychometric function imply the presence and partial transfer of recalibration, to a reliable extent for SoA (and also reliably on average across both tasks; simple main effect *d* = 0.38, *p* = 0.017). All other interactions were not significant for central tendency (τ).

The pattern was different for the width of the psychometric function, which had a group-mean average across all conditions of 311 ms (figure 3b; interquartile range 124 ms) suggesting that participants translated a fairly broad range of subjectively experienced asynchronies into ‘yes’ responses. As illustrated in figure 3d, lag adaptation affected the width of the psychometric function marginally differently depending on the task (two-way interaction *d* = 0.25, *p* = 0.053). There was a non-significant reduction for the simultaneity judgement (SJ) task (orange bars; simple main effect −20 ms, *d* = 0.22, *p* = 0.094) switching to a non-significant increase when SoA was being assessed (purple bars; +13 ms, *d* = 0.11, *p* = 0.297). Auditory tests yielded generally wider windows compared with visual ones (main effect of test +36 ms, *d* = 0.40, *p* ≤ 0.001). Further main effects and interactions were not significant.

Because our model-fitting procedure revealed evidence for a challenging posterior likelihood surface, we explored how sensitive our findings were to a possible local (as opposed to global) maximum likelihood fit, and present this analysis in the electronic supplementary material (Validation of approach to parameter recovery). This analysis suggests that the adaptation effects we can be most confident about are the main effect of adaptation on central tendency (*τ*), extending to three of four combinations of task and sensory modality, and the interaction of adaptation with sensory modality (figure 3c). These are also the most critical effects for testing our hypotheses. We therefore focus our discussion on these effects. Slopes of the left and right flanks of the psychometric functions, which are not typically presented as dependent variables in studies of recalibration, are also presented in the electronic supplementary material (Additional analyses).

## 4. Discussion

We investigated possible differences in the sensorimotor temporal recalibration of time and perceived agency. We assumed that temporal recalibration of perceived agency results from one or a combination of two mechanisms. The first relies on a comparator process that compares the predicted sensory feedback of the action with the actual feedback. The second is driven by a supramodal mechanism in which recalibration of perceived agency occurs for action–feedback events independent of the sensory feedback’s identity, i.e. an action’s learned sensory effect, as long as causality can be established. Here, we asked to what extent prediction of action–feedback identity plays a role in the recalibration of perceived agency. To this end, participants performed button presses that led to the occurrence of a Gabor patch, either immediately after the button press or following a 150 ms lag, inducing adaptation. After the adaptation phase, we presented button press–Gabor patch pairs with variable RSAs and asked participants to judge either the synchrony of the two events or the SoA, or more precisely, authorship over the occurrence of the Gabor patch via the button press. Importantly, we examined whether this recalibration would transfer to another sense by replacing visual stimuli with auditory tones on test trials in a separate block. Overall, we found recalibration of synchrony as well as perceived agency when a systematic lag was introduced between the action–feedback pair, and transfer of this recalibration from vision to audition for agency judgements. However, adaptation delay interacted with the sensory feedback modality at test, suggesting transfer was incomplete. We discuss the findings in detail below.

Considering adaptation effects within the visual modality, we observed a shift in the central tendency for synchrony judgements with adaptation delay, indicating recalibration of perceived synchrony towards more stimulus-lagging RSAs under systematic exposure to a lag (which appears as a rightward shift in our figures). Under our model, central tendency represents the midpoint between two decision criteria for ‘synchronous’ responses, the first when action follows and the second when action leads the sensory feedback. This kind of dependent variable is accepted as a primary measure of temporal recalibration [65]. A similar pattern was observed for agency judgements in which the central tendency of the perceived agency curve shifted towards the right, consistent with the adaptation delay. These results indicate recalibration of synchrony as well as SoA in the presence of systematic action–feedback delays, and are in line with previous work on temporal recalibration of action–feedback events [3,4,6,49–51] and SoA [43].

Our findings also demonstrate transfer of recalibration from vision to audition for agency judgements. Cross-modal recalibration of synchrony between action–feedback pairs has been shown [4,6,45,49–51]. These studies also indicate weaker cross-modal recalibration compared with recalibration within the same sensory modality, and therefore, potentially smaller effect sizes for this effect. For this reason, we recruited a larger number of participants than has been previously implemented, aiming for better estimates for cross-modal transfer effects. Despite a trend towards partial transfer, we did not find evidence for cross-modal transfer of synchrony in a sample size larger than those used in previous studies [6,50,51].

Importantly, we showed for the first time that recalibration of perceived agency can also transfer across senses. In order to determine whether SoA is inferred either as a result of a supramodal clock that recalibrates agency judgements based on timing or via action–feedback identity derived from a comparator process, we asked which of these mechanisms contributes to perceived agency recalibration. For this, we specifically looked for cross-modal transfer of recalibration for judgements of agency. Transfer of perceived agency recalibration from vision to audition supports the former mechanism, namely the supramodal clock in accordance with the causal relationship between actions and their sensory feedback reflected in their temporal order. The transfer effect also speaks in favour of multisensory integration of related signals originating from a common cause [19]. Our results point to the role of a generalized temporal clock rather than exact sensory feedback-identity in inferring and adapting SoA. They are therefore in line with previous work emphasizing causality, potentially via multisensory cue integration processes, as the mechanism driving perceived agency [19,42,48]. Our findings highlight the role of temporal order on recalibration of agency and support the existence of a supramodal clock that is independent of sensory feedback modality. Similar to subjective timing of action–feedback events, recalibration of perceived agency can help to compensate sensorimotor delays and/or integrate multisensory inputs arising from one’s own action.

Despite a somewhat similar adaptation profile for synchrony and agency judgements, we found a significant shift in central tendency with adaptation delay in the auditory modality only for agency. This might indicate higher flexibility in adapting perceived control over the events associated with one’s own action than merely their timing. Alternatively, perceived control may boost the temporal integration of action–feedback events. Either way, this result is in line with previous work highlighting the importance of SoA over learned action–feedback associations [13,42]. A general mechanism that transfers across modalities might have functional advantages over more specific forms of adaptation if the perturbations that require compensation tend to effect multiple types of actions and their associated sensations. One example of such a perturbation would be bodily growth. On the other hand, sensory feedback modality can still play a role in recalibrating SoA, similar to temporal recalibration of action–feedback events and its transfer [49,51,52]. For example, Arikan *et al*. [51] showed cross-modal transfer from motor–visual to motor–auditory temporal recalibration, but not the other way around. They explain these findings by pointing to the dominant role of audition in temporal recalibration as well as the intrinsic connection between motor and auditory processing in judging time [66–69]. Although we failed to replicate transfer of synchrony in our study, one open question is whether agency judgements in the temporal domain exist for motor–visual events (transfer from vision to audition) and not for motor–auditory events (transfer from audition to vision). Future studies should therefore investigate whether sensory feedback modality influences recalibration of perceived agency.

Our study has some limitations. For example, we fitted all of our data with a model of the synchrony-judgement task, which somewhat presupposes that agency is inferred in broadly the same way as synchrony. Clearly, a model based on categorizing the perceived timing between events does not model all potential inputs into a decision about perceived agency. Indeed, we rejected SoA data from a single participant who almost never reported a SoA (while often reporting a sensation of synchrony) so was probably influenced by a non-temporal cue or belief. The mechanisms involved in both synchrony and SoA decisions are doubtless more complex than our simple model has assumed. However, while the exact model parameters we estimated (and their meaning in underlying cognitive terms) are debatable, we suspect that different approaches used to summarize our data would have given rise to a substantially identical interpretation, i.e. that recalibration occurred for SJs and SoA and transferred to button press–auditory feedback conditions, at least for SoA.

A second limitation concerns the potential involvement of auditory imagery in judging synchrony and SoA. In our study, both adaptation and test phases involved pressing the button at a certain rate, which was established with a metronome prior to the experimental blocks. In this sense, our experimental paradigm resembles tapping paradigms in which the action of tapping is synchronized with a metronome, although our task did not require finger flexion/extension and the auditory feedback from the button press was masked by the white noise [68]. Still, the participants may have been encouraged to use some form of auditory imagery throughout the experiment. This could induce neural entrainment to the imagined sequence, modulating attention and affecting the judgements in turn [70,71]. We have no reason to assume that any such auditory imagery can account for differences across conditions as it was present in both baseline and lagged blocks. Nevertheless, the role of auditory imagery on time perception cannot be entirely discounted. One way to eliminate auditory imagery is to utilize experimental paradigms that do not require rhythmic movements such as rapid recalibration paradigms in which recalibration is induced on a trial-by-trial basis without a dedicated adaptation phase [72]. Rapid recalibration paradigms can also address whether adaptation is modulated by previous exposure, i.e. temporal distance between actions and feedback in previous trials, and whether its transfer takes places rather automatically for synchrony and agency judgements.

Some studies have suggested a crucial role for comparator processes in establishing SoA [27–29], whereas others have indicated a complex interplay between sensorimotor and cognitive mechanisms giving rise to perceived agency [48,73,74]. A well-known account by Synofzik *et al*. [25] proposes two distinct levels of SoA: a lower, pre-reflective level highlighted by an implicit sense of being in control (feeling of agency), and a higher, belief-like level underlined by an explicit sense of being in control (judgement of agency). Judgements of agency are found to be less sensitive to action–feedback mismatches, and are thought to rely on a combination of sensorimotor cues and cognitive processes such as prior beliefs and postdictive inferences [73,75,76]. On the other hand, feeling of agency may be less susceptible to learned action–feedback relations, and can rely more on higher-order processes [13,42]. Nevertheless, it should be kept in mind that we measured explicit SoA. Thus, our findings can provide evidence for explicit agency judgements contributing to the recalibration of perceived agency. Future studies should investigate the exact contribution of the comparator process and the supramodal clock on recalibration of both implicit and explicit SoA.

## 5. Conclusion

In summary, this study has provided a large-sample replication of sensorimotor temporal recalibration of perceived synchrony, but not of its cross-modal transfer from vision to audition. Moreover, we additionally replicated temporal recalibration of the SoA and demonstrated for the first time partial cross-modal transfer. This novel result implies that recalibration of perceived agency is derived from a supramodal clock that that adapts the perceived action–event temporal order, rather than the match between the sensory feedback and the predictions of a modality specific and temporally tuned forward model.

## Data Availability

The data and analysis scripts are available at [77]. Additional analyses supporting this article have been uploaded as part of the electronic supplementary material [78].
